# Care-seeking and treatment pathways of multidrug-resistant tuberculosis patients: an analysis of real-world data from regional health information system in Ningbo City in Eastern China

**DOI:** 10.1080/07853890.2025.2496405

**Published:** 2025-04-21

**Authors:** Bin Chen, Yang Li, Ying Peng, Lin Zhou, Xin-yi Chen, Yan-li Ren, Dan Luo, Yu-xiao Ling, Yi-qing Zhou, Jian-min Jiang, Biao Xu

**Affiliations:** a Department of Tuberculosis Control and Prevention, Zhejiang Provincial Center for Disease Control and Prevention, Hangzhou, People’s Republic of China; b School of Public Health, Hangzhou Normal University, Hangzhou, People’s Republic of China; c Songzi Center for Disease Control and Prevention, Jingzhou, People’s Republic of China; d School of Public Health, Hangzhou Medical College, Hangzhou, People’s Republic of China; e School of Public Health, Health Science Center, Ningbo University, Ningbo, People’s Republic of China; f Key Laboratory of Vaccine, Prevention and Control of Infectious Disease of Zhejiang Province, Zhejiang Provincial Center for Disease Control and Prevention, Hangzhou, People’s Republic of China; g Department of Epidemiology, School of Public Health, Fudan University, Shanghai, People’s Republic of China; h Key Laboratory of Health Technology Assessment, National Health Commission of the People’s Republic of China (Fudan University), Shanghai, People’s Republic of China

**Keywords:** Multidrug-resistant tuberculosis, patient pathway analysis, real-world, care seeking, ningbo

## Abstract

**Objectives:**

The patient pathway of multidrug-resistant tuberculosis (MDR-TB) in China is an essential but not well-studied area. This study aimed to understand the alignment between patient-initiated care-seeking demands for MDR-TB and the availability of diagnostic and treatment services in Ningbo, a city in eastern China, using patient pathway analysis (PPA).

**Methods:**

We collected the diagnostic and treatment data of 240 patients with MDR-TB in Ningbo from 2015 to 2019. Using patient pathway analysis, we matched the medical data of patients from different medical institutions and mapped their care pathways to illustrate their access to medical services.

**Result:**

Our study indicated that the proportion of patients with MDR-TB who chose non-TB-designated medical institutions (55%) was higher than those who chose TB-designated medical institutions (45%) at their initial visit. An estimated 69% of patients with MDR-TB patients received initial TB screening services during their first visit. In this study, 47% of patients needed to visit 4–7 medical institutions to be diagnosed with MDR-TB. Overall, 80% (*n* = 192) of patients were diagnosed with MDR-TB within four visits, while 13% (*n* = 30), 4% (*n* = 10), and 3% (*n* = 8) of patients were not diagnosed at the fourth visit and remained at level 2, 1, and 0 medical institutions, respectively.

**Conclusion:**

The care-seeking pathway of patients with MDR-TB in Ningbo is complex. This indicates room for improvement in local diagnosis and referral services. There is a need to promote the deployment of MDR-TB screening, diagnosis, and treatment services at lower-level institutions.

## Introduction

Globally, an estimated 10.6 million people developed tuberculosis (TB), and 410 000 people (95% confidence interval (CI): 370 000–450 000) developed multidrug-resistant or rifampicin-resistant TB (MDR/RR-TB) in 2022 [1]. MDR-TB is a significant public health concern in China due to its unfavorable treatment outcomes and heavy disease burden [2]. The diagnosis and treatment of patients with MDR-TB are challenging due to numerous factors. For example, the diagnosis of MDR-TB through traditional phenotypic (or culture-based) methods is time-consuming, and Drug Susceptibility Testing (DST) is not widely available in many areas. MDR-TB treatment is difficult because the second-line TB drugs are mostly expensive and toxic, and MDR-TB treatment generally takes long time [3]. Inadequate diagnostic and treatment resources contribute to the continued spread and mortality of MDR-TB [4]. Balanced planning for MDR-TB service demand and delivery is crucial, yet it is often underestimated and poorly understood [5].

According to the World Health Organization (WHO), only 81.07% (13,250/16,343) of patients diagnosed with MDR-TB in China were detected and received treatment [6]. Of those treated, only 67.66% (8960/13,250) completed the course or were cured [6]. These unfavorable treatment outcomes indicate that the quality of service for patients with drug-resistant TB in China is unsatisfactory and there is still progress to be made across the TB care cascade. The treatment regimen for MDR-TB is 9–24 months, longer than the regimen for drug-susceptible TB [7]. This extended treatment period makes adherence difficult for patients with MDR-TB. When patients refuse or discontinue treatment due to various factors, such as long duration of treatment, adverse drug reactions (ADRs) and financial hardship [8], it leads to patient loss, significant gaps in the treatment cohort [9], and an increased risk of social transmission.

Several tools have been developed to assess the challenges in accessing TB medical services, such as the Onion Model [10], patient care cascades [11], MATCH approach [12], and Finding all the Missing Persons [13]. The Onion Model and patient care cascades emphasize breaking down the healthcare service process to identify weak points throughout the entire chain, from disease screening, diagnosis, treatment and rehabilitation. MATCH approach and Finding all the Missing Persons focus on identifying key barriers that lead to patients being ‘lost to follow-up’ or experiencing interruptions in care. Recently, the patient pathway analysis (PPA) was recommended by the Lancet Commission [14]. PPA helps to understand the alignment between patient care-seeking and service availability and to identify measures to optimize timely diagnosis and treatment services [15]. Most PPA studies apply existing institutional service data or secondary national/local survey data to analyze patient service pathways; however, few studies have examined patient pathways through real-world digital records [16–19].

Two previous studies have analyzed and described the TB patient pathway in China. One developed an algorithmic approach to analyze and interpret patient-level routine data from the local Health Insurance Database [20]. This study demonstrated that longitudinal analysis of routine individual-level healthcare data can be used to generate a detailed picture of TB care-seeking pathways. The other was conducted for newly bacteriologically diagnosed patients with PTB and focused on the delay in diagnosing patients with TB [21]. This study found that patients who went to low-level facilities initially had a higher risk of long diagnostic delay (LDD) [21]. However, these studies did not use PPA to assess the alignment of TB or MDR-TB service delivery with patient care-seeking patterns [22]. The PPA was designed to assess the alignment between TB care-seeking patterns and the availability of TB services. The PPA could be a valuable planning and programming tool to ensure that diagnostic and treatment services are available to patients where they seek care.

This study aims to use PPA to present the care service experiences of patients with MDR-TB using data collected from a local real-world health information platform. Our goal is to better understand the alignment between care-seeking and the availability of diagnostic and treatment services for patients with MDR-TB in Ningbo, China, and to provide evidence-based recommendations to optimize the spatial allocation of TB medical resources and improve service for patients with MDR-TB.

## Methods

Zhejiang Province, a developed province in eastern China, has an annual reported pulmonary tuberculosis (PTB) incidence rate of approximately 30/100 000(9). Although the incidence of PTB in this province has decreased in recent years, the current reduction rate may not be adequate to reach future demands of the End TB target set by the WHO that ‘the incidence rate of TB should be reduced to below 10 cases per 100,000 population by 2035’. In Ningbo, a prefecture-level city in Zhejiang Province, the annual PTB incidence rate is at the average provincial level [23]. At that time, medical resources were limited and unevenly allocated. Ningbo city has attracted 4.5 million migrants from different regions in 2020. The immigrant populations in Ningbo show relatively a high prevalence of DR-TB, which highlights the need for clinical control of TB [24]. We chose Ningbo for our study because of its mature and reliable regional health information platform, which records patient consultation, diagnosis, and treatment information from all medical institutions in the city [25]. This platform also covers the local patient with MDR-TB consultations and treatment information, offering a valuable opportunity to understand the real-world care-seeking and treatment experiences of patients with MDR-TB. Given that the platform was initially established in 2014 and to eliminate the potential influence of the COVID-19 pandemic on the diagnosis and treatment of MDR-TB patients, this study utilized data from the period of 2015 to 2019 for analysis, thereby enhancing the robustness of the study.

The PPA methodology introduced by Hanson et al. [26] was employed to evaluate the alignment between patient care-seeking and the availability of TB diagnostic and treatment services. In this study, identification (ID) information of registered patients with drug-resistant TB in Ningbo from 2015 to 2019 was collected using Tuberculosis Information Management System (TBIMS). The ID numbers of the patients were then matched with the local health information platform of Ningbo City to obtain the care-seeking data.

**Figure 1.**
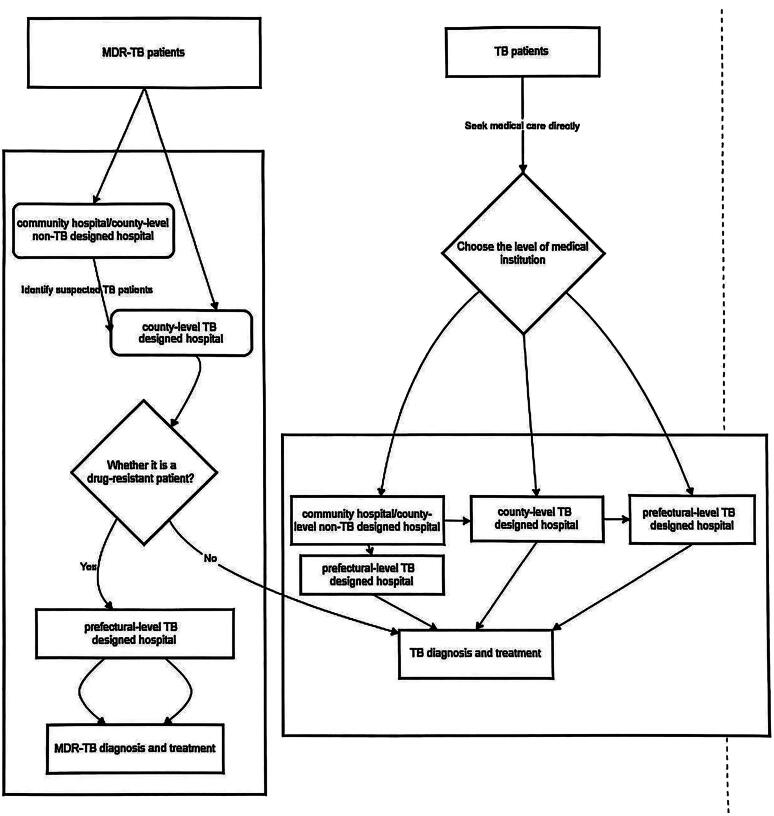
Flowchart of care-seeking pathways for common TB and MDR-TB patients.

China implements a hierarchical diagnosis and treatment system, health system is divided into community-level, county-level and prefectural-level medical institutions. According to the hierarchical diagnosis and treatment system, patients prefer to seek initial care at Low-level medical institutions. However, Low-level medical institutions had insufficient TB laboratory and radiographic equipment. High-level medical institutions had a low rate of utilization of TB laboratory equipment [21]. The MDR-TB diagnosis and treatment service system in China operates through prefectural and county-level medical institutions. Patients with TB can seek diagnosis and treatment in any TB-designated hospital. In contrast, patients with MDR-TB are screened in county-level TB-designated hospitals and diagnosed and treated in prefectural TB-designated hospitals. Non-TB-designated medical institutions at the county level or below identify suspected patients with TB and refer them to county-level TB-designated hospitals. Upon confirmation of RIF-resistant TB, the patient is transferred to a prefectural TB-designated hospital for further management.(See Figure 1)

To comprehensively understand the experience and flow of patients with MDR-TB in medical institutions in Ningbo, we graded and classified the medical institutions based on their service functions and hierarchical levels. Each institution was categorized as either a TB-designated or non-TB-designated medical institution and assigned levels ranging from 0–2. At the same time, the level 0, 1, 2 medical institution was defined as the low-level, intermediate-level high-level medical institution, respectively. (See supplemental data)

A detailed map of the different categories and levels of medical institutions is shown in Table 1. All of the above-mentioned medical institutions are public institutions.

**Table 1.** Categories and levels of medical institutions in ningbo.

Institution	Institution Category	Institution Level
Community/village health service stations	Non-TB designated Medical Institutions	Level 0
Township health service centers	Non-TB designated Medical Institutions	Level 0
County level hospitals	Non-TB designated Medical Institutions	Level 1
County level hospitals	TB-designated Medical Institutions	Level 1
Prefectural level hospitals	Non-TB designated Medical Institutions	Level 2
Prefectural level hospitals	TB-designated Medical Institutions	Level 2

## Data analysis and calculation procedure

We utilized a PPA model to estimate the likelihood of patients with MDR-TB receiving appropriate care services along the medical pathway. We were informed by previous research, and derived from existing literature. Earlier PPA studies relied on TB epidemiological surveys, WHO reports [27], patient registries [20], and national TB control program data [22]. While previous PPA studies used seven indicators [15], we developed eight indicators to evaluate better the service accessibility and adaptability of the diagnosis and treatment processes within the unique service model in China. (see Supplementary data)

1. Percentage of first visiting institutions

This indicator measures the percentage of patients with MDR-TB who first visited a specific level (L0–L2) and category (TB-designated or non-TB-designated) medical institution due to suspected TB symptoms.

1. Percentage of institutions with TB screening capacity

This indicator reflects the proportion of healthcare institutions where patients visited first that can conduct basic TB screening tests such as chest radiography or preliminary sputum testing.

1. Access to primary TB screening services

This indicator reflects the proportion of patients attending an institution with basic TB screening capacity. This indicator can be interpreted as the likelihood of patients accessing primary TB screening services during their first visit.

1. TB diagnosis and treatment service coverage

This indicator measures the proportion of health institutions qualified to provide TB diagnosis and treatment services.

1. Access to TB diagnosis and treatment services

This indicator denotes the proportion of patients attending medical institutions qualified to provide TB diagnosis and treatment services during their first visit. This indicator can be interpreted as the likelihood that a patient will have access to TB diagnosis and treatment services on their first visit to a doctor.

1. Coverage of drug-resistant TB diagnosis and treatment services

This indicator denotes the proportion of medical institutions qualified to provide diagnosis and treatment services for drug-resistant TB.

1. Access to drug-resistant TB diagnosis and treatment services

This measures the likelihood of patients accessing diagnosis and treatment services for drug-resistant TB on their first visit to qualified medical institutions.

1. Final treatment outcomes

This indicator represents the proportion of treatment outcomes achieved by all patients with MDR-TB included in the analysis after completing their treatment period.

## Patient service pathway visualization

The final visualization of patient pathways was created using Adobe Illustrator, with Tableau serving as the primary software for completing the analysis and driving the underlying PPA visualization. The Tableau workbook, which is freely downloadable (https://www.tableau.com, accessed April 19, 2024), provides users with the necessary underlying structure for designing a PPA figure. Data integration from an Excel worksheet (Microsoft, Redmond, WA, USA) enabled the structuring and display of the PPA figure, where all eight indicators are represented as columns. Different bar charts and colors in the figure represent different indicators and their respective values across the columns.

The distribution of clinical visits among MDR-TB patients could reflect the number of visits to medical institutions of different levels and categories, thereby highlighting the complexity of the care pathway for patients with MDR-TB. The analysis of medical institutions visited by the patients was presented using a stacked column chart, mainly using Tableau, to visualize the final data. Operations involved collecting data from Excel tables containing diagnosis and treatment information of all patients with MDR-TB, utilizing drag-and-drop features to create views, applying colors and filters to deepen emphasis, and exporting chart files.

The first four patient visits were selected for visualization of patient flow in this study. Informed by previous research, Sankey diagrams were used to effectively illustrate the flow of patients through different healthcare pathways [21]. The flow of patient visits was visualized using a Sankey diagram, mainly created in Adobe Illustrator. RAW Graphs is a data visualization tool used to complete Sankey diagrams. The primary operations included loading data, selecting charts, associating data items with visualization variables, customizing chart configuration items, and exporting chart files.

## Ethics approval

The data in this study were derived from TBIMS and regional health platform. The Ethics Review Committee of the Zhejiang Provincial Center for Disease Control and Prevention reviewed the study project and issued an ethical review waiver for informed consent. All information related to the identities of the research subjects was deleted prior to the commencement of the study. Patient privacy was protected and not disclosed in this study. Hereby, we declare that our study adheres to the Declaration of Helsinki.

## Results

### Patient demographics

A total of 329 patients with drug-resistant TB were identified in the system and matched with the local health information platform of Ningbo City. Patients were excluded due to not being classified under the standard definition of MDR-TB. Subsequently, patients with no medical records and incomplete information (*n* = 36), those with mono-resistance to rifampicin or isoniazid, extensive drug resistance, and other drug-resistant strains (*n* = 50), as well as patients with non-TB mycobacterium (*n* = 3) were excluded from the analysis. This process resulted in effective data from 240 patients with MDR-TB being utilized for the PPA. Basic demographic and clinical information of the patients are shown in Table 2.

**Table 2.** Demographic and treatment details of patients with MDR-TB in ningbo City (2015 to 2019).

Variant		n	%
Gender	Male	171	71
	Female	69	29
Age	Age 30 and below	35	15
	31–60	144	60
	Over 60 years	61	25
Classification of treatment	Initial treatment	96	40
	Retreatment	144	60
Diagnostic method	Molecular biological testing	113	47
	Culture	127	53
Time of registration	2015	38	16
	2016	25	10
	2017	54	23
	2018	59	24
	2019	64	27
Levels of first visiting institution after symptom onset	Prefectural level	97	40
	County level	86	36
	Community level	57	24
Levels of institution for treatment after diagnosis of MDR	Prefectural level	184	77
	County level	3	1
	Community level	0	0
	Untreated	53	22

### MDR-TB patient pathway and service alignment

**Figure 2.**
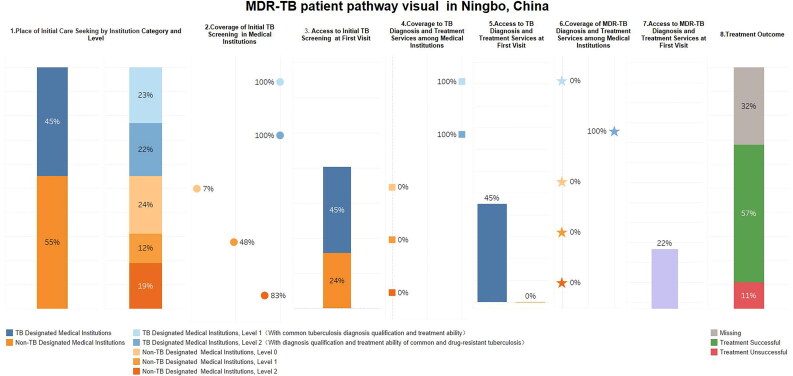
MDR-TB patient pathway visual in Ningbo, China. The patient-pathway analysis visual describes several potential steps along a patient’s journey for TB care. The first column shows where patients initiate their care-seeking journey across different categories and levels of the healthcare system. Column 2 estimates the percentage of medical institutions at each category and level that have initial TB screening services in the institution. Column 3 estimates the likelihood that a patient will start their care-seeking journey in an institution that has TB screening tools. This is calculated by multiplying the proportion of patients who seek care at each category and level of medical institution (column 1) by the initial TB screening coverage rate of corresponding category and level (column 2). The fourth column shows the coverage of TB diagnosis and treatment services at each category and level of the health system. Similar to column 3, column 5 estimates the likelihood of a patient started their care-seeking journey in an institution that has TB diagnosis and treatment services. This is calculated by multiplying care seeking at each category and level of medical institution (column 1) by the coverage rate of TB diagnosis and treatment services of corresponding category and level (column 4). Column 6 shows the coverage of MDR-TB diagnosis and treatment services at each category and level of the health system. Similar to column 5, column 7 estimates the likelihood of a patient started their care-seeking journey in an institution that has MDR-TB diagnosis and treatment services. The final column shows the treatment outcome of MDR-TB patients in this study.

Based on the selection criteria, 240 patients with MDR-TB were included in this PPA. Figure 2 illustrates the patient pathway, revealing that during their initial visit, a higher proportion of patients with MDR-TB chose non-TB-designated medical institutions (55%) compared to TB-designated medical institutions (45%). The coverage rates of TB initial screening services in non-TB designated medical institutions at level 0, level 1, and level 2 were 7%, 48%, and 83% respectively. While the coverage rates of TB initial screening services in TB designated medical institutions at level 1 and level 2 were both 100%. It was estimated that 69% (*n* = 165) of the 240 patients with MDR-TB received an initial TB screening service during their first visit. The coverage rates of TB diagnosis and treatment services in non-TB designated medical institutions were all zero. In contrast, the coverage rates of TB diagnosis and treatment services in TB designated medical institutions at level 1 and level 2 were both 100%. Among those screened, approximately 45% (*n* = 108) received TB diagnosis and treatment services for TB during their first visit. The coverage rate of MDR-TB diagnosis and treatment services in TB designated medical institutions at Level 2 was 100%, while it was zero at all other medical institutions. Approximately 22% (*n* = 53) of patients with MDR-TB received diagnosis and treatment services for drug-resistant TB, including screening for drug resistance during their first visit.

Ultimately, 57% (*n* = 137) of patients with MDR-TB were successfully treated, 11% (*n* = 27) were not successfully treated, and 32% (*n* = 76) were lost to follow-up during the diagnosis and treatment process.

### The number of medical institutions visited by MDR-TB patients

**Figure 3.**
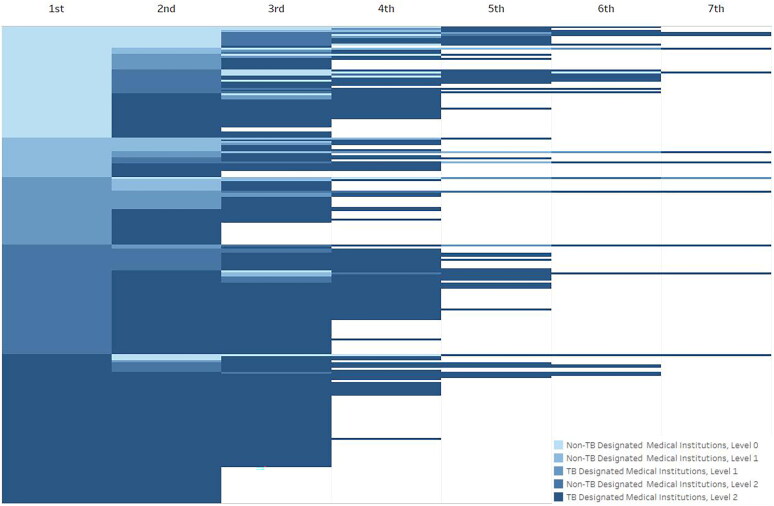
The medical institutions visited by patients with MDR-TB patients before diagnosis. Different gradations of color represent different levels and categories of medical institutions. Each medical visit among 240 participants stratified by the categories and levels of health facilities. The top texts refer to the times of medical visits. Each row presents one case.

Figure 3 illustrates the distribution of clinical visits among patients with MDR-TB in Ningbo from 2015–2019, ranging from 1–7 visits (median 3.00; Interquartile range (IQR) 3.00–4.00). Of these visits, 53% (*n* = 128) resulted in MDR-TB diagnosis through 1–3 medical institutions, while 38% (*n* = 91) of diagnosis of MDR-TB occurred through 4–5 medical institutions. Notably, 9% (*n* = 21) of patients required visits at 6–7 medical institutions before being diagnosed with MDR-TB.

In terms of initial medical institutions visited by patients, the distribution across different categories was as follows: non-TB designated medical institutions at level 0 were 23% (*n* = 56), non-TB designated medical institutions at level 1 were 13% (*n* = 31), TB designated medical institutions of level 1 comprised 23% (*n* = 54), non-TB designated medical institutions at level 2 constituted 19% (*n* = 46), and TB designated medical institutions at level 2 accounted for 22% (*n* = 53).

Among the patients who initially visited non-TB-designated medical institutions at level 0, 18 (32%) patients were diagnosed with MDR-TB after visiting 1–3 medical institutions, while 25 (45%) and 13 (23%) patients required visits to 4–5 and 6–7 medical institutions, respectively. Among the patients who had their first visit at non-TB designated medical institutions at level 1, 12 (39%), 16 (51%), and 3 (10%) patients were diagnosed with MDR-TB after 1–3, 4–5, and 6–7 medical institution visits, respectively. Patients who initially visited TB-designated medical institutions of level 1, 38 (70%), 14 (26%), and 2 (4%) patients were diagnosed through 1–3, 4–5, and 6–7 medical institutions, respectively. For patients who first visited non-TB-designated medical institutions at level 2, 15 (32%), 29 (63%), and 2 (4%) patients were diagnosed through 1–3, 4–5, and 6–7 medical institutions, respectively. Lastly, among patients directly visiting optimal MDR-TB designated medical institutions at level 2 for their first visit, 45 (85%) patients were diagnosed through 1–3 medical institutions, and 7 (13%) patients were diagnosed through 4–5 medical institutions.

### Service flow among different institutions for MDR-TB patients

**Figure 4.**
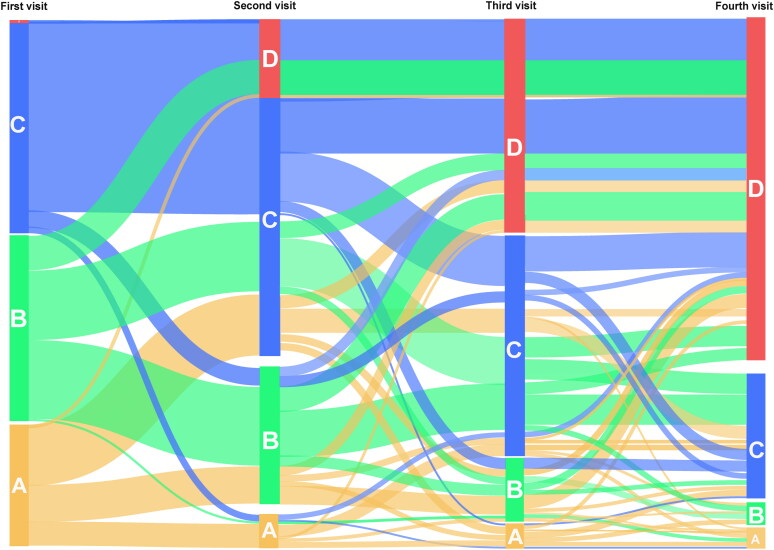
Sankey diagram of the patient’s visits. A-orange part refers to patients who first visited community-level medical institutions (medical institutions at level 0). B-green part, refers to patients who first visited county-level medical institutions (medical institutions at level 1, including TB-designated and non-TB-designated medical institutions). C-blue part, refers to patients who first visited prefectural-level medical institutions (medical institutions at level 2, including TB-designated and non-TB-designated medical institutions). D-Red part, refers to patients who first diagnosed with MDR-TB at prefectural TB-designated medical institutions.

The Sankey diagram of patient visits (Figure 4) illustrates that 80% (*n* = 192) of patients were diagnosed with MDR-TB within four visits. The remaining 13% (*n* = 30), 4% (*n* = 10), and 3% (*n* = 8) of the patients were not diagnosed by their fourth visit and remained in level 2, 1, and 0 medical institutions, respectively.

Among the confirmed cases, 45% (*n* = 87) of the patients first visited level 2 (prefectural level) medical institutions (Blue part), with 78 patients (90%) being diagnosed after visiting level 2 medical institutions 2–4 times. Among the confirmed cases, 37% (*n* = 71) of the patients first visited level 1(county level) medical institutions (Green part). Of these, 35 patients (50%) were transferred from level 1 to level 2 medical institutions 1–3 times before being diagnosed, and 23 patients (32%) visited a level 2 medical institution after two visits to level 1 medical institutions before being diagnosed. The remaining 18% (*n* = 34) of patients with confirmed MDR-TB first visited level 0 (community level) medical institutions (Orange part). Of these, 17 (50%) patients visited level 0 to level 2 medical institutions 1–3 times before diagnosis, and 12 (35%) patients visited level 1 medical institutions 1–2 times after visiting a level 0 medical institution before being transferred to level 2 medical institution where they were finally diagnosed (Figure 4).

Among the 13%(*n* = 30) of patients who remained undiagnosed at level 2 medical institutions by the fourth visit, 40% (*n* = 12) of the patients first visited a level 0 medical institution (community level), and four patients were transferred from level 0 to level 2 medical institutions three times without being diagnosed. Additionally, 20% (*n* = 6) of these patients visited level 2 medical institutions four times without diagnosis.

Of the 8% (*n* = 18) of patients who remained undiagnosed at level 0 and 1 medical institutions by their fourth visit, most (*n* = 10) first visited a level 0 medical institution. Six of these patients continued to visit level 0 and 1 medical institutions without being transferred to level 2 medical institutions.

## Discussion

The PPA method, invented by Hanson et al. evaluates the alignment between patient care-seeking and medical service availability [26]. Patients with MDR-TB experience longer and more complex diagnostic and treatment pathways compared to those with drug-susceptible TB [28,29]. PPA can evaluate the alignment between care-seeking and the availability of TB services, helping to identify healthcare supply and demand gaps and optimize medical resource allocation. To our knowledge, this is the first study on the pathway of patients with MDR-TB using real-world digital data in China.

Significant differences exist in the accessibility of TB diagnosis and treatment services across countries. In Pakistan, Indonesia, and the Philippines, most patients are diagnosed or treated for TB in public or formal private medical institutions [16,18,30]. In contrast, patients with TB in China are primarily diagnosed and treated in designated TB hospitals at the county and prefectural levels. Data show that the hierarchical level of medical institutes where patients initially sought care in China is higher than in Kenya, Cameroon, and South Africa [19,27,31]. The differences between China and other countries arise from variations in healthcare system design, economic development levels, and policy priorities. China has implemented a hierarchical diagnosis and treatment system, where centralized management ensures high-quality medical care but may limit accessibility. In contrast, other countries often adopt a mixed public-private model, where decentralized services enhance coverage but may encounter technical challenges. While seeking care at higher-level medical institutions may lead to early detection and better treatment for patients with MDR-TB, it also entails longer service distances and higher costs.

In this study, it was estimated that approximately 70% of patients with MDR-TB received the initial screening service for TB during their first visit, and 57% of patients with MDR-TB were successfully treated. This indicates that patients with MDR-TB have early access to TB screening services, which facilitate early case detection. However, the successful treatment rates for drug-resistant TB remained suboptimal. In comparison, a study in Cameroon estimated that 9% of patients accessed TB diagnostic and treatment services at their initial point of care, with 44% successively treated [19]. In a PPA study in South Africa, approximately 53% of patients with TB successfully completed treatment, with 5% not receiving diagnostic services and 17% not successfully completing treatment [27]. In Kenya, 26% of patients began treatment in the formal private sector, and 15% sought treatment from informal sector providers [31]. These findings suggest that, similar to other countries, there are still weak links in the diagnostic and treatment services, leading to poor outcomes in patients with MDR-TB.

In this study, nearly 23% of the patients chose low-level medical institutions for their first visit, with 45% and 23% requiring visits to 4–5 and 6–7 medical institutions before being diagnosed with MDR-TB, respectively. Approximately 22% of people started their first visit at high-level TB-designated medical institutions, with only 13% needing visits to 4–5 medical institutions for diagnosis. Patients who first visited high-level medical institutions had fewer visits than those who started at low-level medical institutions. This highlights the necessity to enhance the deployment of MDR-TB screening, diagnosis, and treatment services at primary healthcare institutions, while simultaneously improving clinicians’ awareness and diagnostic capabilities regarding tuberculosis [32]. We also found that patients had different flow directions before being diagnosed with MDR-TB. Among the patients diagnosed after four visits and first visited a low-level medical institution, 35% were transferred 1–2 times to intermediate-level medical institutions before finally being transferred to a high-level medical institution, where they were diagnosed. Among the patients who remained at high-level medical institutions after the fourth visit and were not diagnosed, 20% of them visited high-level medical institutions four times but remained undiagnosed, highlighting the complexity of the care pathway for patients with MDR-TB.

In a Chinese study of 260 individuals who initially visited L2–L3 facilities, 236 continued to seek care at these levels and were diagnosed there [21]. Nineteen patients repeatedly visited both low- and high-level hospitals before being diagnosed at L2–3 facilities, and five were diagnosed at L0–L1 facilities. Patients who initially visited L2–L3 facilities had fewer clinical visits compared to those who started at L0–L1 facilities [21]. Similar to other studies [20,21], this study indicates that the care-seeking pathways of patients with MDR-TB in Ningbo are complex and highlight the need for improved local referral processes.

Centralizing TB diagnosis and treatment in TB-designated hospitals in China is crucial for patient management and fee waivers. However, this centralization may reduce the awareness of clinicians about TB in non-TB-designated hospitals [21]. This indicates that training for healthcare workers (HCWs) is essential to enhance the quality of diagnostic and treatment services. A study in Tanzania demonstrates that training and mentoring HCWs at decentralized sites improved their knowledge and skills in DR-TB care, leading to favorable interim and final patient outcomes despite health system challenges [33]. In contrast to the situation in China, a study in KwaZulu-Natal suggests that decentralized care for MDR-TB patients yields better outcomes compared to centralized hospital care [34]. Additionally, a meta-analysis reveals that decentralized care is more likely to result in successful treatment outcomes and appears to be either cost-neutral or cost-saving relative to centralized approaches [35]. From 2015 to 2019, hospitals in Ningbo City introduced GeneXpert MTB/RIF technology, which can significantly shorten the diagnosis time of patients with drug-resistant TB [36]. However, at that time, medical resources were limited and unevenly allocated. Despite its benefits, the technology was only available in TB-designated hospitals and was costly, limiting patient accessibility.

Our study had certain limitations. First, the analysis explored the alignment between initial care-seeking and the availability of TB services for patients with MDR-TB, assuming that the TB disease was symptomatic. This assumption is increasingly contested as some patients with TB do not report symptoms [37]. However, the PPA model in our study reflects the medical service process after the occurrence of symptoms, which is more common. Second, few patients with MDR-TB may also visit the medical institutions out of Ningbo prefecture in the pathway and we could not collect these data by using the local information system, which introduces a confounding factor that warrants further investigation. These could be potential areas of expansion of PPA methods in future research. Third, some data like socioeconomic position, educational level, comorbid diseases, severity of disease and distance to hospital/health center were hard to acquire, which may hindered the comprehensive analysis. These variables may be the focus for future research. Fourth, the 2015–2019 data cut-points may provide a limitation to contemporary practice.

## Conclusion

Our study provides a new perspective for evaluating the quality of health services based on PPA, allowing for a deeper understanding of the alignment of patient flow and the supply of healthcare services. Using real-world medical data, we found that nearly half of patients with MDR-TB in Ningbo City initially visited a TB-designated medical institution, and the majority received initial TB screening service on their first visit. Although patients with MDR-TB had access to fundamental TB services early in their illness, their pathway through the healthcare system was relatively complex, indicating room for improvement in the local diagnosis and referral service system.

Patients with MDR-TB in Ningbo who started their care at grass-root non-TB designated institutions tended to have more visits to different institutes and longer pathways later before being diagnosed and treated. This suggests a need to promote the deployment of MDR-TB screening, diagnosis, and treatment services at lower-level institutions, improve the awareness and diagnostic capacity of clinicians for TB, and shorten the time and distance of patient visits. Improving these aspects can enhance the overall efficiency and effectiveness of MDR-TB patient care.

## Supplementary Material

Figure legends - IANN-2024-4022.R1.docx

Medical institutions classification.docx

The calculation method of the indicators in the PPA model.docx

## Data Availability

The datasets used and analyzed in this study are available from the corresponding author (Jianmin Jiang) upon reasonable request.

## Supplementary data

Supplementary materials are available at Annals of Medicine online. Consisting of data provided by the authors to benefit the reader, the posted materials are not copyedited and are the sole responsibility of the authors, so questions or comments should be addressed to the corresponding author.
