# Supplementary material for: Care-seeking and treatment pathways of multidrug-resistant tuberculosis patients: an analysis of real-world data from regional health information system in Ningbo City in Eastern China
Source: Ann Med. 2025 Apr 21;57(1):2496405. doi: 10.1080/07853890.2025.2496405 (PMC12016273; doi:10.1080/07853890.2025.2496405)
Supplement: Figure legends - IANN-2024-4022.R1.docx [file IANN_A_2496405_SM9532.docx]

**Figure 1.** Flowchart of care-seeking pathways for common TB and MDR-TB patients.

**Figure2.** MDR-TB patient pathway visual in Ningbo, China. The patient-pathway analysis visual describes several potential steps along a patient’s journey for TB care. The first column shows where patients initiate their care-seeking journey across different categories and levels of the healthcare system. Column 2 estimates the percentage of medical institutions at each category and level that have initial TB screening services in the institution. Column 3 estimates the likelihood that a patient will start their care-seeking journey in an institution that has TB screening tools. This is calculated by multiplying the proportion of patients who seek care at each category and level of medical institution (column 1) by the initial TB screening coverage rate of corresponding category and level (column 2). The fourth column shows the coverage of TB diagnosis and treatment services at each category and level of the health system. Similar to column 3, column 5 estimates the likelihood of a patient started their care-seeking journey in an institution that has TB diagnosis and treatment services. This is calculated by multiplying care seeking at each category and level of medical institution (column 1) by the coverage rate of TB diagnosis and treatment services of corresponding category and level (column 4). Column 6 shows the coverage of MDR-TB diagnosis and treatment services at each category and level of the health system. Similar to column 5, column 7 estimates the likelihood of a patient started their care-seeking journey in an institution that has MDR-TB diagnosis and treatment services. The final column shows the treatment outcome of MDR-TB patients in this study.

**Figure 3.** The medical institutions visited by patients with MDR-TB patients before diagnosis. Different gradations of color represent different levels and categories of medical institutions. Each medical visit among 240 participants stratified by the categories and levels of health facilities. The top texts refer to the times of medical visits. Each row presents one case.

**Figure 4.** Sankey diagram of the patient's visits. A-Orange part refers to patients who first visited community-level Medical Institutions (medical institutions at level 0). B-Green part, refers to patients who first visited county-level Medical Institutions (medical institutions at level 1, including TB-designated and non-TB-designated Medical Institutions). C-Blue part, refers to patients who first visited prefectural-level Medical Institutions (medical institutions at level 2, including TB-designated and non-TB-designated Medical Institutions). D-Red part, refers to patients who first diagnosed with MDR-TB at prefectural TB-designated Medical Institutions.
