# Supplementary material for: Care-seeking and treatment pathways of multidrug-resistant tuberculosis patients: an analysis of real-world data from regional health information system in Ningbo City in Eastern China
Source: Ann Med. 2025 Apr 21;57(1):2496405. doi: 10.1080/07853890.2025.2496405 (PMC12016273; doi:10.1080/07853890.2025.2496405)
Supplement: Medical institutions classification.docx [file IANN_A_2496405_SM9531.docx]

**Medical institutions classification**

**Level 0 (L0): Refers to the most basic community-based care level. Level 0 services include basic patient triage, health information registration, primary prevention, and care. Services are provided by medical or paramedical staff with basic training. No TB laboratory testing is available, but L0 staff may serve as treatment follow-up supporters for patients with TB. L0 institutions in Ningbo include township health service centers and community/village health service stations.**

**Level 1 (L1): Refers to institutions providing medical services at the county level. Basic TB screening and diagnostic services, including sputum microscopy and essential drugs, are provided by TB-designated hospitals at these levels. In Ningbo, L1 institutions refer to county-level hospitals, encompassing both TB-designated and non-TB-designated hospitals.**

**Level 2 (L2): Refers to institutions providing more advanced diagnosis and treatment services at prefectural levels. L2 institutions commonly offer more specialized diagnostics and treatment options in outpatient and inpatient settings. TB-designated hospitals at the L2 level provide access to specialized TB doctors and sophisticated diagnostic and treatment services, particularly for patients with MDR-TB. In Ningbo, there are mainly prefectural-level hospitals, encompassing both TB- and non-TB-designated hospitals.** **A detailed map of the different categories and levels of medical institutions is shown in Table 2.**
