# Supplementary material for: Care-seeking and treatment pathways of multidrug-resistant tuberculosis patients: an analysis of real-world data from regional health information system in Ningbo City in Eastern China
Source: Ann Med. 2025 Apr 21;57(1):2496405. doi: 10.1080/07853890.2025.2496405 (PMC12016273; doi:10.1080/07853890.2025.2496405)
Supplement: The calculation method of the indicators in the PPA model.docx [file IANN_A_2496405_SM9530.docx]

**The calculation method of the indicators in the PPA model**

1. Percentage of first visiting institutions

The numerator of this indicator is the number of patients arriving at a certain level or category of healthcare institution for the first time, and the denominator is the total number of patients included in the analysis.

(2) Percentage of institutions with TB screening capacity

This indicator is calculated according to the different levels or categories of medical institutions. The numerator is the number of different levels or categories of medical institutions capable of providing primary TB screening services, and the denominator is the total number of medical institutions at the corresponding level or category that patients visited first.

(3) Access to primary TB screening services.

It is calculated by multiplying indicator (1) by indicator (2) at different levels and categories and then adding the product.

(4) TB diagnosis and treatment service coverage

The numerator is the number of institutions at various levels or categories qualified to provide these services as determined by the local health administration department. The denominator is the total number of medical institutions at the corresponding level or category.

(5) Access to TB diagnosis and treatment services.

Calculations can be performed at different levels and categories of institutions. It is calculated by multiplying indicator (1) by indicator (4) across different levels and categories and then adding the product.

(6) Coverage of drug-resistant TB diagnosis and treatment services

The numerator is the number of institutions at different levels or categories qualified for the diagnosis and treatment of drug-resistant TB, which is determined by the health administration department at the provincial level. The denominator is the total number of health institutions at the corresponding level or category.

(7) Access to drug-resistant TB diagnosis and treatment services

It is calculated by multiplying indicator (1) by indicator (6) across different levels and categories of institutions and then adding the product.

(8) Final treatment outcomes

This indicator represents the proportion of treatment outcomes achieved by all patients with MDR-TB included in the analysis after completing their treatment period.
